# Supplementary material for: The Small RNA Universe of Capitella teleta
Source: Front Mol Biosci. 2022 Feb 25;9:802814. doi: 10.3389/fmolb.2022.802814 (PMC8915122; doi:10.3389/fmolb.2022.802814)
Supplement: Supplementary file 1 [file DataSheet1.ZIP › Supplement/known/CAPTEscaffold_339_17080.pdf]

Provisional ID	:	CAPTEscaffold_339_17080
Score total	:	603941.3
Score for star read(s)	:	3.9
Score for read counts	:	603930.8
Score for mfe	:	2
Score for randfold	:	1.6
Score for cons. seed	:	3
Total read count	:	1184596
Mature read count	:	1184590
Loop read count	:	0
Star read count	:	6

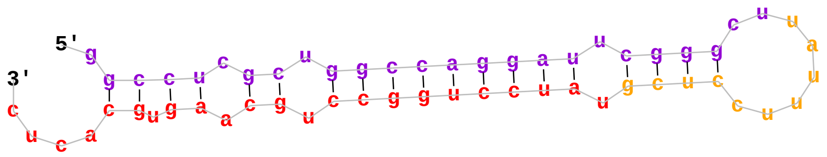

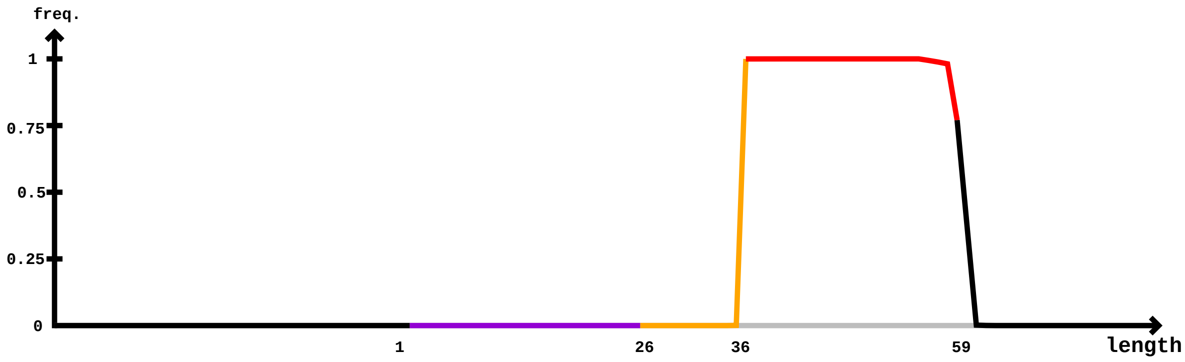

Star Mature

```
5'- auacaaacgcuacuugaauugucguuuccgaaucaggccucgcuggccaggauucgggcuuauuuccucguauccuggccugcaagugcacucauucggacgaccuuaucucc  -3'  obs
    auacaaacgcuacuugaauugucguuuccgaaucaggccucgcuggccaggauucgggcuuauuuccucguauccuggccugcaagugcacucauucggacgaccuuaucucc       exp
    auacaaacgcuacuugaauugucguuuccgaaucaggccucgcuggccaggauucgggcuuauuuccucguauccuggccugcaagugcacucauucggacgaccuuaucucc       known
    ..................((((..(((((((.((((((.((.(((((((((.((((........)))).))))))))).)).)).)).)).))))))))))).........    reads  mm  sample

    ..................................aggccucgcuggccaggauucg.........................................................    1      0   seq
    ...................................ggccucgcuggccaggauucgg........................................................    1      0   seq
    ...................................ggccucgcuggccaggaGucgggcu.....................................................    1      1   seq
    ...................................ggccucgcuggccaggauucgggcu.....................................................    3      0   seq
    ...................................................................ucguauccuggccugcaagugc........................    1      0   seq
    ....................................................................cguauccuggccugcaagugc........................    3      0   seq
    .....................................................................guauccuggccugcaagu..........................    3      0   seq
    .....................................................................guauccuggccugcaagugG........................    1      1   seq
    .....................................................................guauccuggccugcaagugc........................    127    0   seq
    .....................................................................guauccugUccugcaagugc........................    1      1   seq
    .....................................................................guaucUuggccugcaagugca.......................    1      1   seq
    .....................................................................guauccuggccugcaagugca.......................    11     0   seq
    .....................................................................guauccuggccugcaagugcac......................    9      0   seq
    .....................................................................guauccuggccugAaagugcacu.....................    1      1   seq
    .....................................................................Cuauccuggccugcaagugcacu.....................    14     1   seq
    .....................................................................guauccuggccugcaaCugcacu.....................    1      1   seq
    .....................................................................Uuauccuggccugcaagugcacu.....................    1      1   seq
    .....................................................................gCauccuggccugcaagugcacu.....................    1      1   seq
    .....................................................................guauccuggccugcaagugcacu.....................    400    0   seq
    .....................................................................guauccAggccugcaagugcacu.....................    1      1   seq
    .....................................................................Cuauccuggccugcaagugcacuc....................    41     1   seq
    .....................................................................guauccuggccugcaagugcacuc....................    28     0   seq
    .....................................................................guauccuggccugcaagugcacuU....................    3      1   seq
    .....................................................................Uuauccuggccugcaagugcacuc....................    1      1   seq
    .....................................................................guauccuggccugcaagugcacucU...................    13     1   seq
    ......................................................................uauccuggccugcaagug.........................    10     0   seq
    ......................................................................Gauccuggccugcaagugc........................    5      1   seq
    ......................................................................uauccuggccugcaagugG........................    6      1   seq
    ......................................................................uaucUuggccugcaagugc........................    6      1   seq
    ......................................................................uauUcuggccugcaagugc........................    2      1   seq
    ......................................................................uaucGuggccugcaagugc........................    1      1   seq
```

Star **Mature**

auacaaacgcuacuugaauugucguuuccgaaucaggccucgcuggccaggauucgggcuuauuuccucguauccuggccugcaagugcacucauucggacgaccuuaucucc			
....uauccuggccugcaaUugc........................	1	1	seq
....uauccuggccugcUagugc........................	1	1	seq
....uauccugAccugcaagugc........................	2	1	seq
....uauccuggccugcGagugc........................	1	1	seq
....uauccAggccugcaagugc........................	4	1	seq
....uauccuggccuUcaagugc........................	1	1	seq
....uauccuggcAugcaagugc........................	1	1	seq
....uauccuggAcugcaagugc........................	1	1	seq
....Aauccuggccugcaagugc........................	29	1	seq
....uauccuggccugcaaguUc........................	3	1	seq
....uauccuggccugcaagGgc........................	1	1	seq
....uauccuggccugcaagugc........................	6406	0	seq
....uaAccuggccugcaagugc........................	12	1	seq
....uauccuggccugcaaguCc........................	1	1	seq
....uauAcuggccugcaagugc........................	2	1	seq
....uauccuggcUugcaagugc........................	1	1	seq
....uauccuAgccugcaagugc........................	3	1	seq
....uNuccuggccugcaagugc........................	1	1	seq
....uGuccuggccugcaagugc........................	1	1	seq
....uauccuggccugcaagAgc........................	2	1	seq
....uauccugCccugcaagugc........................	1	1	seq
....uauccuggccugcaGgugc........................	3	1	seq
....uauccuggUcugcaagugc........................	1	1	seq
....uauccuggccugcaagugU........................	1	1	seq
....uauccuggccugAaagugc........................	2	1	seq
....uauccuggccugcaagugA........................	8	1	seq
....uauccuggccCgcaagugc........................	1	1	seq
....uauccuggccAgcaagugc........................	2	1	seq
....Cauccuggccugcaagugc........................	1	1	seq
....uauGcuggccugcaagugc........................	2	1	seq
....uaGccuggccugcaagugca.......................	1	1	seq
....uauccuggccugcaagugcG.......................	21	1	seq
....uaCccuggccugcaagugca.......................	1	1	seq
....uaucUuggccugcaagugca.......................	3	1	seq
....uauccuggccugcaagugcU.......................	2	1	seq
....uGuccuggccugcaagugca.......................	1	1	seq
....uaucGuggccugcaagugca.......................	1	1	seq
....uauccuggcAugcaagugca.......................	2	1	seq
....uauccuggccAgcaagugca.......................	2	1	seq
....uauccuggccugcaagugca.......................	7016	0	seq
....uauccuggccugGaagugca.......................	1	1	seq
....uauccugAccugcaagugca.......................	1	1	seq
....uauccuggAcugcaagugca.......................	2	1	seq
....uauccuggccugcaaguAca.......................	1	1	seq
....Aauccuggccugcaagugca.......................	27	1	seq
....uauccuggcUugcaagugca.......................	2	1	seq
....uauccuUgccugcaagugca.......................	2	1	seq
....uauccuggccugcaaUugca.......................	2	1	seq
....uauccuggccugUaagugca.......................	3	1	seq
....uauccuggccugcaagAgca.......................	4	1	seq
....uauccuAgccugcaagugca.......................	5	1	seq
....uauccuggccuUcaagugca.......................	1	1	seq
....uauccuggccugcaagCgca.......................	1	1	seq
....uauccAggccugcaagugca.......................	6	1	seq
....uauGcuggccugcaagugca.......................	1	1	seq
....uauccuggccugcGagugca.......................	2	1	seq
....uauccuggccugcaGgugca.......................	1	1	seq
....uauccuggccCgcaagugca.......................	1	1	seq
....uauccuggccugAaagugca.......................	1	1	seq
....uauccuggccugcaagugAa.......................	2	1	seq
....Gauccuggccugcaagugca.......................	2	1	seq
....uaAccuggccugcaagugca.......................	16	1	seq
....Cauccuggccugcaagugca.......................	2	1	seq
....uauAcuggccugcaagugca.......................	2	1	seq
....uauccuggccuCcaagugca.......................	1	1	seq
....uauccuggccugcaagugcaA......................	2	1	seq
....uauccuggccugcaagugcac......................	8250	0	seq
....uauccuggccugGaagugcac......................	1	1	seq
....Aauccuggccugcaagugcac......................	35	1	seq
....uauAcuggccugcaagugcac......................	3	1	seq

Star **Mature**

```
auacaaacgcuacuugaauugucguuuccgaaucaggccucgcuggccaggauucgggcuuauuuccucguauccuggccugcaagugcacucauucggacgaccuuaucucc
......................................................................uaGccuggccugcaagugcac......................	1	1	seq
......................................................................uaucGuggccugcaagugcac......................	1	1	seq
......................................................................uauccuggccAgcaagugcac......................	4	1	seq
......................................................................uauccAggccugcaagugcac......................	5	1	seq
......................................................................uauccuggccugcaUgugcac......................	1	1	seq
......................................................................Gauccuggccugcaagugcac......................	3	1	seq
......................................................................uauccuggccugcaagAgcac......................	5	1	seq
......................................................................uauccuggccugcaagGgcac......................	1	1	seq
......................................................................uauccuggccugUaagugcac......................	2	1	seq
......................................................................uauccuggcUugcaagugcac......................	1	1	seq
......................................................................uauccuggccugcaaguUcac......................	2	1	seq
......................................................................uauccuUgccugcaagugcac......................	1	1	seq
......................................................................uauccuggccuCcaagugcac......................	1	1	seq
......................................................................uauccuggccugcaagugcUc......................	2	1	seq
......................................................................uauccuggcAugcaagugcac......................	2	1	seq
......................................................................uauUcuggccugcaagugcac......................	1	1	seq
......................................................................uauccuggccugcaagugGac......................	1	1	seq
......................................................................uaAccuggccugcaagugcac......................	15	1	seq
......................................................................uaucCggccugcaagugcac......................	1	1	seq
......................................................................uauccuggccCgcaagugcac......................	1	1	seq
......................................................................uauccuggccugcaagugcGc......................	2	1	seq
......................................................................uauccuggccugAaagugcac......................	3	1	seq
......................................................................Cauccuggccugcaagugcac......................	1	1	seq
......................................................................uauccugCccugcaagugcac......................	1	1	seq
......................................................................uaucAuggccugcaagugcac......................	3	1	seq
......................................................................uaucUuggccugcaagugcac......................	4	1	seq
......................................................................uauccuggUcugcaagugcac......................	2	1	seq
......................................................................Nauccuggccugcaagugcac......................	2	1	seq
......................................................................uauccuAgccugcaagugcac......................	3	1	seq
......................................................................uauccuggGcugcaagugcac......................	2	1	seq
......................................................................uauccuggccugcaaguAcac......................	1	1	seq
......................................................................uauccuggccugcUagugcac......................	2	1	seq
......................................................................uauccuggccugcaagugcaU......................	6	1	seq
......................................................................uauccuAgccugcaagugcacu.....................	135	1	seq
......................................................................uaNccuggccugcaagugcacu.....................	2	1	seq
......................................................................uauccAggccugcaagugcacu.....................	145	1	seq
......................................................................uauccuggccugcaCgugcacu.....................	1	1	seq
......................................................................uauccuggccugcaagugcCcu.....................	2	1	seq
......................................................................uauUcuggccugcaagugcacu.....................	31	1	seq
......................................................................uaucGuggccugcaagugcacu.....................	18	1	seq
......................................................................Aauccuggccugcaagugcacu.....................	1006	1	seq
......................................................................uauccuggccugcaagAgcacu.....................	101	1	seq
......................................................................uauccugCccugcaagugcacu.....................	12	1	seq
......................................................................uauccuggccugcaagGgcacu.....................	11	1	seq
......................................................................uauGcuggccugcaagugcacu.....................	9	1	seq
......................................................................uauccuggccugcaaguUcacu.....................	12	1	seq
......................................................................uauccuggGcugcaagugcacu.....................	13	1	seq
......................................................................uauccuggccugcaaAugcacu.....................	17	1	seq
......................................................................uauccuggccuNcaagugcacu.....................	1	1	seq
......................................................................uauccuggccuAcaagugcacu.....................	46	1	seq
......................................................................uauccuggccugcaagugcGcu.....................	41	1	seq
......................................................................uauccuggcGugcaagugcacu.....................	3	1	seq
......................................................................uauccuggccugcUagugcacu.....................	20	1	seq
......................................................................uauccuggccugcaGgugcacu.....................	38	1	seq
......................................................................uauccuggccuCcaagugcacu.....................	21	1	seq
......................................................................uauccuggccugcaagugcacu.....................	245296	0	seq
......................................................................uaAccuggccugcaagugcacu.....................	544	1	seq
......................................................................uauccuggccugcaaguCcacu.....................	13	1	seq
......................................................................uNuccuggccugcaagugcacu.....................	2	1	seq
......................................................................uGuccuggccugcaagugcacu.....................	41	1	seq
......................................................................Gauccuggccugcaagugcacu.....................	140	1	seq
......................................................................uauccuggccugcaagugcaGu.....................	21	1	seq
......................................................................uauccuggccugcaaCugcacu.....................	3	1	seq
......................................................................uauccuggccugcaagugcUcu.....................	32	1	seq
......................................................................uauccugNccugcaagugcacu.....................	1	1	seq
......................................................................uauccuggcUugcaagugcacu.....................	70	1	seq
......................................................................uauccuggccugcCagugcacu.....................	8	1	seq
......................................................................uauccuggccugGaagugcacu.....................	20	1	seq
......................................................................uaucUuggccugcaagugcacu.....................	184	1	seq
......................................................................uauccuggccugcaagCgcacu.....................	24	1	seq
```

Star **Mature**

auacaaacgcuacuugaauugucguuuccgaaucaggccucgcuggccaggauucgggcuuauuuccucguauccuggccugcaagugcacucauucggacgaccuuaucucc			
....uauccuggccugcaUgugcacu.....................	12	1	seq
....uUuccuggccugcaagugcacu.....................	17	1	seq
....uauccuggccugcGagugcacu.....................	64	1	seq
....uauAcuggccugcaagugcacu.....................	72	1	seq
....uauccuCgccugcaagugcacu.....................	12	1	seq
....uauccugAccugcaagugcacu.....................	60	1	seq
....Nauccuggccugcaagugcacu.....................	67	1	seq
....uauccuggcAugcaagugcacu.....................	88	1	seq
....uauccuggccugcaagugcaNu.....................	2	1	seq
....uauccuggccGgcaagugcacu.....................	6	1	seq
....uauccuggccugcaagugUacu.....................	70	1	seq
....uauccuggccugcaagugcacA.....................	79	1	seq
....uauccuggccugUaagugcacu.....................	48	1	seq
....uaucAuggccugcaagugcacu.....................	40	1	seq
....uauccuggAcugcaagugcacu.....................	56	1	seq
....uauccuUgccugcaagugcacu.....................	13	1	seq
....uauccuggccugcNagugcacu.....................	1	1	seq
....uauccuggccugcaagugcaUu.....................	19	1	seq
....uaCccuggccugcaagugcacu.....................	33	1	seq
....uauccuggccugcaagugcacC.....................	24	1	seq
....uauccuggccAgcaagugcacu.....................	65	1	seq
....uaGccuggccugcaagugcacu.....................	2	1	seq
....uauccuggccugcaagugGacu.....................	27	1	seq
....uauccuggccuUcaagugcacu.....................	21	1	seq
....uauccugUccugcaagugcacu.....................	15	1	seq
....Cauccuggccugcaagugcacu.....................	29	1	seq
....uauccuggccugcaaUugcacu.....................	5	1	seq
....uauccGggccugcaagugcacu.....................	4	1	seq
....uauccuggccugcaagugcacG.....................	174	1	seq
....uauccuggUcugcaagugcacu.....................	55	1	seq
....uauccNggccugcaagugcacu.....................	3	1	seq
....uauccuggccugcaaguAcacu.....................	33	1	seq
....uauccuggccCgcaagugcacu.....................	77	1	seq
....uauccuggccugcaagugAacu.....................	50	1	seq
....uauccCggccugcaagugcacu.....................	31	1	seq
....uauccuggccugAaagugcacu.....................	94	1	seq
....uauccuggccugcaagugcaAu.....................	20	1	seq
....Gauccuggccugcaagugcacuc....................	466	1	seq
....uauccuggccugcaagugcacuc....................	757698	0	seq
....uauccuggccuUcaagugcacuc....................	44	1	seq
....uauccuUgccugcaagugcacuc....................	47	1	seq
....uauccuggccugcaaguCcacuc....................	44	1	seq
....uauccugAccugcaagugcacuc....................	141	1	seq
....uauccuggAcugcaagugcacuc....................	144	1	seq
....uauccuggccugcaaCugcacuc....................	29	1	seq
....uauccuggccugUaagugcacuc....................	108	1	seq
....uauccuggccugcaagugcacuN....................	3	1	seq
....uauccuggccuCcaagugcacuc....................	61	1	seq
....uauccuggccugcaagugcaGuc....................	51	1	seq
....uauccuggccugAaagugcacuc....................	226	1	seq
....uauccuggcGugcaagugcacuc....................	22	1	seq
....uauAcuggccugcaagugcacuc....................	210	1	seq
....uauccuggGcugcaagugcacuc....................	34	1	seq
....uauccuggccugcaagugcUcuc....................	91	1	seq
....uauccuAgccugcaagugcacuc....................	499	1	seq
....uauccuggccugcaagugcCcuc....................	14	1	seq
....uaucGuggccugcaagugcacuc....................	38	1	seq
....uUuccuggccugcaagugcacuc....................	52	1	seq
....uauccNggccugcaagugcacuc....................	3	1	seq
....uauccuggccugcaagugcacAc....................	194	1	seq
....uauccuggccuAcaagugcacuc....................	152	1	seq
....Nauccuggccugcaagugcacuc....................	220	1	seq
....uauccuggUcugcaagugcacuc....................	111	1	seq
....uauccuggcNugcaagugcacuc....................	2	1	seq
....Cauccuggccugcaagugcacuc....................	102	1	seq
....uauccuggccugcaaUugcacuc....................	23	1	seq
....uaNccuggccugcaagugcacuc....................	5	1	seq
....uaCccuggccugcaagugcacuc....................	90	1	seq
....uauccuggccugcUagugcacuc....................	79	1	seq
....uauccuggccugcGagugcacuc....................	190	1	seq

Star Mature

auacaaacgcuacuugaauugucguuuccgaaucaggccucgcuggccaggauucgggcuuauuuccucguauccuggccugcaagugcacucauucggacgaccuuaucucc			
....uauccuggccugcaagugcGcuc....................	167	1	seq
....uauccuNgccugcaagugcacuc....................	2	1	seq
....uauccuggccugcaaguUcacuc....................	48	1	seq
....uauccuggccugGaagugcacuc....................	65	1	seq
....uauccugCccugcaagugcacuc....................	68	1	seq
....uCuccuggccugcaagugcacuc....................	4	1	seq
....uauccuggccugcaagCgcacuc....................	110	1	seq
....uauccuggccugcaagugcaAuc....................	99	1	seq
....uauccuCgccugcaagugcacuc....................	68	1	seq
....uauccuggccugcaagAgcacuc....................	360	1	seq
....uauccuggccGgcaagugcacuc....................	24	1	seq
....uauGcuggccugcaagugcacuc....................	32	1	seq
....uauUcuggccugcaagugcacuc....................	190	1	seq
....uauccuggccugcaagugcacuG....................	34	1	seq
....uauccuggccugcaagugcaNuc....................	1	1	seq
....uauccGggccugcaagugcacuc....................	14	1	seq
....uauccuggccugcaagugcaUuc....................	65	1	seq
....uauccuggccugcaagNgcacuc....................	1	1	seq
....uaucAuggccugcaagugcacuc....................	131	1	seq
....uauccuggcUugcaagugcacuc....................	147	1	seq
....uauccuggccAgcaagugcacuc....................	244	1	seq
....uauccuggccugcaagGgcacuc....................	18	1	seq
....uauccuggccCgcaagugcacuc....................	199	1	seq
....uauccuggccugcaaAugcacuc....................	47	1	seq
....uauccAggccugcaagugcacuc....................	401	1	seq
....uaAccuggccugcaagugcacuc....................	1303	1	seq
....uauccuggccugcaagugGacuc....................	61	1	seq
....uaGccuggccugcaagugcacuc....................	10	1	seq
....uGuccuggccugcaagugcacuc....................	134	1	seq
....Aauccuggccugcaagugcacuc....................	2799	1	seq
....uauccuggccugcaagugcacGc....................	24	1	seq
....uauccuggcAugcaagugcacuc....................	292	1	seq
....uauccuggccNgcaagugcacuc....................	1	1	seq
....uauccuggccugcaUgugcacuc....................	56	1	seq
....uauccuggccugcCagugcacuc....................	8	1	seq
....uauccuggccugcaagugcacuA....................	435	1	seq
....uauccuggccugcaagugcacuU....................	869	1	seq
....uauccuggccugcaGgugcacuc....................	127	1	seq
....uauccuggccugcaagugAacuc....................	158	1	seq
....uauccuggccugcaaguAcacuc....................	92	1	seq
....uauccugUccugcaagugcacuc....................	65	1	seq
....uauccCggccugcaagugcacuc....................	82	1	seq
....uauccuggccugcaagugcacCc....................	69	1	seq
....uNuccuggccugcaagugcacuc....................	25	1	seq
....uaucUuggccugcaagugcacuc....................	542	1	seq
....uauccuggccugcaagugUacuc....................	73	1	seq
....uauccuggccugcaCgugcacuc....................	2	1	seq
....uauccuggccugcaagugcacuca...................	7981	0	seq
....uauccuggccugcaUgugcacuca...................	2	1	seq
....uauccuggAcugcaagugcacuca...................	3	1	seq
....uauccuggccugcaaAugcacuca...................	2	1	seq
....uauccuggccugGaagugcacuca...................	1	1	seq
....uauccuggccCgcaagugcacuca...................	1	1	seq
....uauccuggccAgcaagugcacuca...................	1	1	seq
....uauccuUgccugcaagugcacuca...................	1	1	seq
....uauccuggccugcaagugcacAca...................	2	1	seq
....Aauccuggccugcaagugcacuca...................	37	1	seq
....uauccuggccugcaagugcacuUa...................	13	1	seq
....uauccuggccugcaagugcacCca...................	1	1	seq
....uauccuggccuCcaagugcacuca...................	1	1	seq
....uaucAuggccugcaagugcacuca...................	2	1	seq
....Cauccuggccugcaagugcacuca...................	2	1	seq
....uaAccuggccugcaagugcacuca...................	8	1	seq
....uauccuggccugUaagugcacuca...................	2	1	seq
....uauccuggccugAaagugcacuca...................	3	1	seq
....uauccuggcAugcaagugcacuca...................	3	1	seq
....uauccuggccugcaagAgcacuca...................	6	1	seq
....uauccuggccugcaagugcacucN...................	5	1	seq
....uauccugUccugcaagugcacuca...................	2	1	seq
....uauccuggccugcaagugcacucC...................	145	1	seq

Star **Mature**

```
auacaaacgcuacuugaauugucguuuccgaaucaggccucgcuggccaggauucgggcuuauuuccucguauccuggccugcaagugcacucauucggacgaccuuaucucc
......................................................................uauccuggccugcaagugcUcuca...................  1       1  seq
......................................................................Gauccuggccugcaagugcacuca...................  6       1  seq
......................................................................uauccuggGcugcaagugcacuca...................  2       1  seq
......................................................................uauccuggcUugcaagugcacuca...................  1       1  seq
......................................................................uauccuggccugcaagugcacucU...................  130571  1  seq
......................................................................uaucUuggccugcaagugcacuca...................  8       1  seq
......................................................................uauccuggccugcGagugcacuca...................  2       1  seq
......................................................................uauAcuggccugcaagugcacuca...................  1       1  seq
......................................................................uaCccuggccugcaagugcacuca...................  2       1  seq
......................................................................uauccuggccugcaagugcacucG...................  176     1  seq
......................................................................uauccuggccugcaagugcacuAa...................  2       1  seq
......................................................................uauccuggccuAcaagugcacuca...................  2       1  seq
......................................................................uauGcuggccugcaagugcacuca...................  1       1  seq
......................................................................uGuccuggccugcaagugcacuca...................  1       1  seq
......................................................................uauccuggccugcaagugcaAuca...................  1       1  seq
......................................................................uauccuggccugcaagugAacuca...................  5       1  seq
......................................................................uauccuggcGugcaagugcacuca...................  2       1  seq
......................................................................uauccuggUcugcaagugcacuca...................  2       1  seq
......................................................................uauccuggccugcaaguAcacuca...................  2       1  seq
......................................................................uauccuggccugcaagugcGcuca...................  3       1  seq
......................................................................uauccugAccugcaagugcacuca...................  2       1  seq
......................................................................uauccuggccugcaagCgcacuca...................  2       1  seq
......................................................................uauccAggccugcaagugcacuca...................  4       1  seq
......................................................................uauccuggccugcaagugcacucCu..................  5       1  seq
......................................................................uauccuggccugcaagugcacuUau..................  3       1  seq
......................................................................Aauccuggccugcaagugcacucau..................  1       1  seq
......................................................................uauccuggccugcaagugcacuAau..................  1       1  seq
......................................................................uauccuggccugcaagugcacucau..................  139     0  seq
......................................................................uauccuggccugcaagugcacucaA..................  27      1  seq
......................................................................uauccuggccugcaagugcacucUu..................  1426    1  seq
......................................................................uauccuggccugcaagugcacucGu..................  3       1  seq
......................................................................uauccuggccugcaagugcacucaAu.................  6       1  seq
......................................................................uauccugAccugcaagugcacucauu.................  1       1  seq
......................................................................uauccuggccugcaagugcacucauu.................  178     0  seq
......................................................................uauccuggccugcaagugcacucaGu.................  1       1  seq
......................................................................uauccuggccugcaagugcacucauA.................  1       1  seq
......................................................................uauccuggccugcaagugcacucUuu.................  254     1  seq
......................................................................uaAccuggccugcaagugcacucauu.................  1       1  seq
......................................................................uauccuggccugcaagugcacucauuU................  2       1  seq
.......................................................................auccuggccugcaagugca.......................  2       0  seq
.......................................................................auccuggccugcaagugcac......................  2       0  seq
.......................................................................Uuccuggccugcaagugcacu.....................  1       1  seq
.......................................................................auccuggccugcaagugcacu.....................  13      0  seq
.......................................................................auccugUccugcaagugcacuc....................  1       1  seq
.......................................................................auccuggccugcaagugcacuc....................  65      0  seq
.......................................................................auUcuggccugcaagugcacuc....................  1       1  seq
.......................................................................Guccuggccugcaagugcacuc....................  1       1  seq
.......................................................................auccuggccugcaagugcacucU...................  3       1  seq
........................................................................uccuggccugcaagugcacu.....................  2       0  seq
........................................................................Gccuggccugcaagugcacuc....................  1       1  seq
........................................................................uccuggccugcaagugcacuc....................  34      0  seq
........................................................................uccuggccugcaagugcacucC...................  1       1  seq
........................................................................uccuggccugcaagugcacucU...................  79      1  seq
.........................................................................ccuggccugcaagugcacu.....................  1       0  seq
.........................................................................ccuggccugcaagugcacuc....................  15      0  seq
..........................................................................cuggccugcaagugcacu.....................  8       0  seq
..........................................................................cuggccugcaagugcacuc....................  50      0  seq
..........................................................................cuggccugcaagugcacuca...................  1       0  seq
..........................................................................cuggccugcaagugcacucU...................  3       1  seq
...........................................................................uggccugcaagugcacuc....................  3       0  seq
```
